# Sphingolipids Are Dual Specific Drug Targets for the Management of Pulmonary Infections: Perspective

**DOI:** 10.3389/fimmu.2017.00378

**Published:** 2017-03-29

**Authors:** Lalita Sharma, Hridayesh Prakash

**Affiliations:** ^1^Laboratory of Translational Medicine, School of Life Sciences, University of Hyderabad, Hyderabad, Telengana, India

**Keywords:** sphingolipids, ceramide, sphingosine kinase-1, macrophages, lungs, *microbes*

## Abstract

Sphingolipids are the major constituent of the mucus secreted by the cells of epithelial linings of lungs where they maintain the barrier functions and prevent microbial invasion. Sphingolipids are interconvertible, and their primary and secondary metabolites have both structural and functional roles. Out of several sphingolipid metabolites, sphingosine-1 phosphate (S1P) and ceramide are central molecules and decisive for sphingolipid signaling. These are produced by enzymatic activity of sphingosine kinase-1 (SK-1) upon the challenge with either biological or physiological stresses. S1P and ceramide rheostat are important for the progression of various pathologies, which are manifested by inflammatory cascade. S1P is a well-established secondary messenger and associated with various neuronal, metabolic, and inflammatory diseases other than respiratory infections such as *Chlamydia pneumoniae, Streptococcus pneumoniae*, and *Mycobacterium tuberculosis*. These pathogens are known to exploit sphingolipid metabolism for their opportunistic survival. Decreased sphingosine kinase activity/S1P content in the lung and peripheral blood of tuberculosis patients clearly indicated a dysregulation of sphingolipid metabolism during infection and suggest that sphingolipid metabolism is important for management of infection by the host. Our previous study has demonstrated that gain of SK-1 activity is important for the maturation of phagolysosomal compartment, innate activation of macrophages, and subsequent control of mycobacterial replication/growth in macrophages. Furthermore, S1P-mediated amelioration of lung pathology and disease severity in TB patients is believed to be mediated by the selective activation or rearrangement of various S1P receptors (S1PR) particularly S1PR2, which has been effective in controlling respiratory fungal pathogens. Therefore, such specificity of S1P–S1PR would be paramount for triggering inflammatory events, subsequent activation, and fostering bactericidal potential in macrophages for the control of TB. In this review, we have discussed and emphasized that sphingolipids may represent effective novel, yet dual specific drug targets for controlling pulmonary infections.

## Introduction

**Figure 1:**
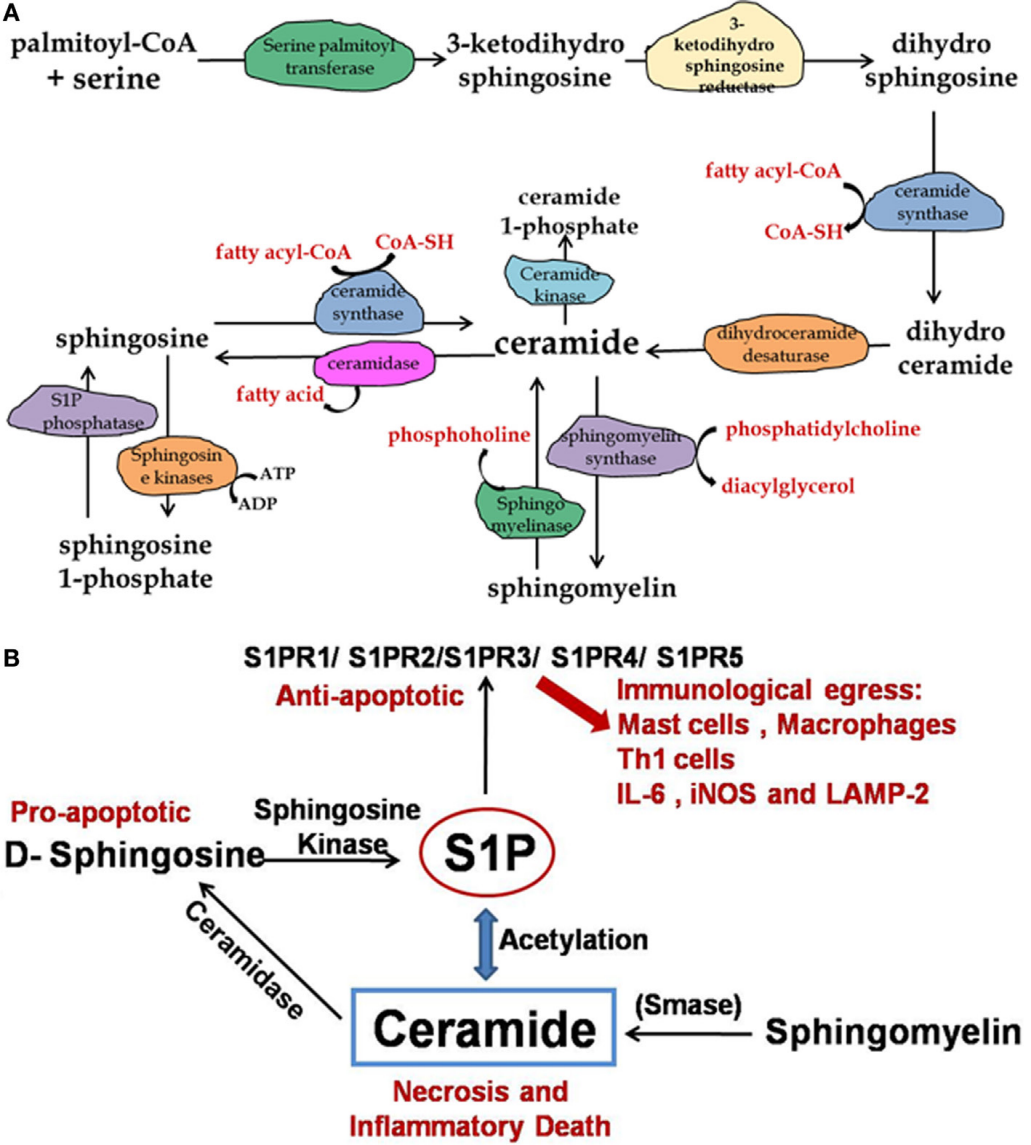
**Pathway for synthesis of ceramide, sphingomyelin, and sphingosine-1 phosphate**. **(A)** The *de novo* synthesis of ceramide starts with palmitoyl-CoA and serine in endoplasmic reticulum. Ceramide is then converted to sphingomyelin, which is the structural component of outer leaflet of plasma membrane. **(B)** Enhanced ceramide concentration in lungs results in inflammation and cell damage therefore dynamic balance of sphingosine/S1P/ceramide is important for pathological manifestation during TB infection.

Sphingolipids are crucial bioactive molecules and involved in several fundamental and pathophysiological processes. A novel therapeutic potential of sphingolipids has been documented for the treatment of asthma, cystic fibrosis, respiratory tract infection, and acute lung injuries (1–3). Sphingolipids are one of the active constituents of the mucus secreted by alveolar epithelium, which protects the lung tissue from invading pathogens. A large number of intermediate metabolites in the mucus are secreted by alveolar epithelium where they act as surfactants and maintain the barrier integrity. One of the important aspects of sphingolipids is their interconvertible nature, which enables them to both integrate and regulate plethora of cellular functions (4, 5) (Figure 1). Among various sphingolipids metabolites, ceramide and sphingosine-1 phosphate (S1P) are the best studied in context of various pathologies. While ceramide and free sphingosine induce cell death and promote sterile inflammation, S1P and ceramide-1-phosphate (C1P) promote cell division and survival and maintain homeostasis (6). Therefore, a fine balance in the level of ceramide and other sphingosine metabolites particularly S1P is critical for cellular homeostasis. S1P initiates its signals *via* G protein-coupled receptors, named as S1P receptors (S1PR). Till date, five different types of S1PR (S1PR1–5) have been discovered, and their temporal expression in cells determines the fate of S1P signaling in various organs. Of five S1PR, receptors 1, 2, and 3 have been studied in context of immune regulation and pathogen control. Interaction of S1P with S1PR1 enhances vascular permeability and T cell egress as seen in many autoimmune and respiratory diseases such as experimental autoimmune encephalomyelitis, airway hyper responsiveness, and pulmonary eosinophil sequestration (7, 8). Pharmacological inhibition of S1PR-1 signaling by FTY-720 (structural analog of sphingosine) also known as Fingolimod has demonstrated that sphingolipids are important drug target for controlling autoimmune disorders (9–11).

**Figure 2:**
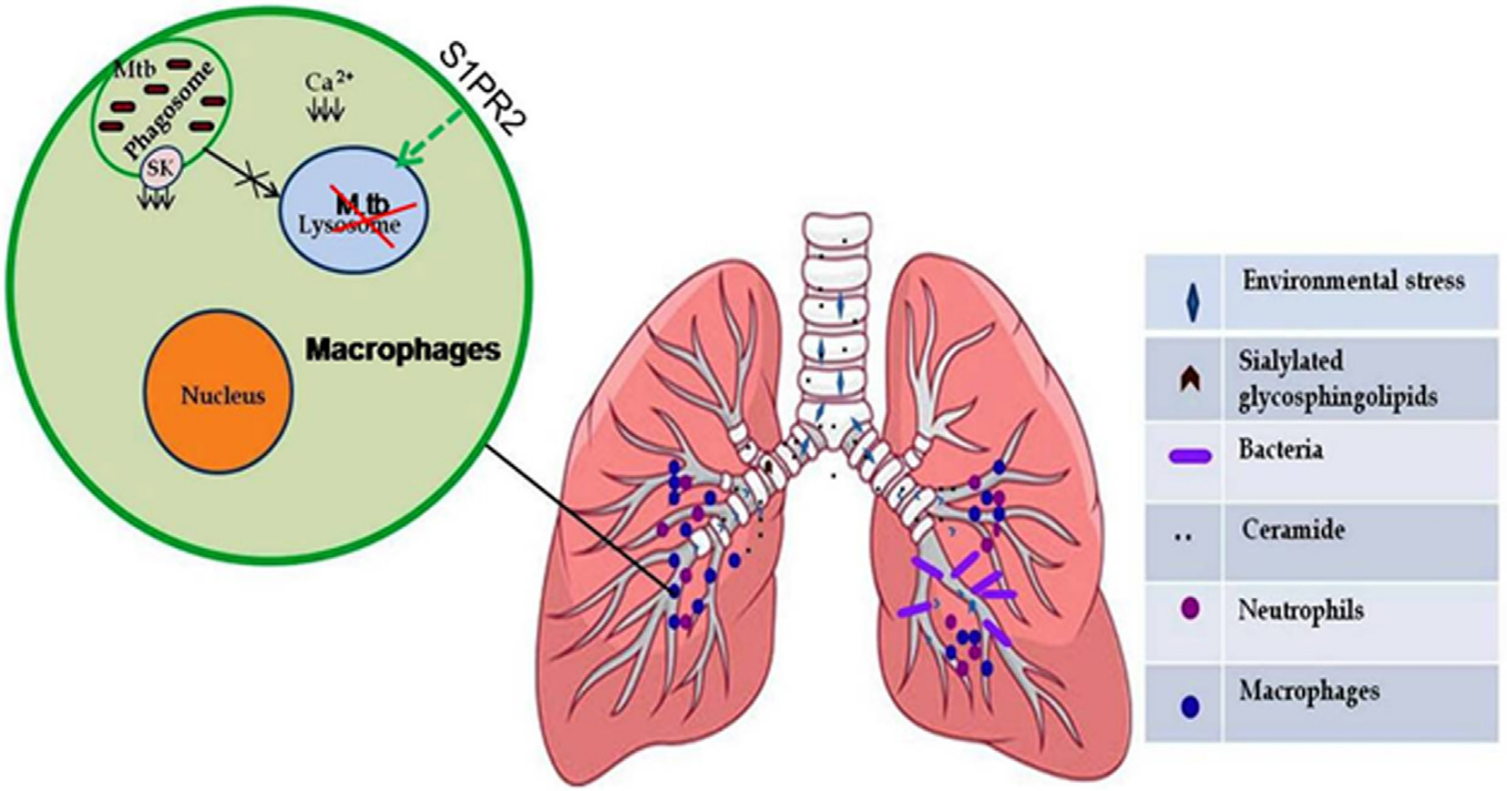
**Sphingolipids (S1P) mediate protective inflammatory response during infection**. Certain environmental stress such as air pollution and respiratory diseases caused by genetic alterations (cystic fibrosis) led to an increase in sialylated glycosphingolipid content in epithelial cell lining of lungs, which serve as receptors for many bacteria (*Mycoplasma pneumonia* and *Pseudomonas aeruginosa*). *Mycobacterium tuberculosis* inhibits the activity of sphingosine kinase in macrophages, which results in decreased intracellular concentration of Ca2+ ions and subsequent phagosome maturation arrest that can be modulated by selective upregulation of S1PR2-associated antimicrobial signaling in the alveolar macrophages.

Three major enzymes have been shown to execute overall sphingolipid metabolisms. These are sphingomyelin synthase/lysase, ceramide synthase/lysase, and sphingosine kinase/lysase. The enzyme most frequently associated with human ailments is acid sphingomyelinase, which remains elevated and contributes to the pulmonary inflammation (1, 12, 13). However, sphingosine kinase-1 (SK-1) and SK-2 have been implicated in immune-cell regulation (14, 15). SK-1 has been shown to mediate mycobacterial infection-induced innate immune response and also capable of controlling mycobacterial infection (16). In the same line, S1P, a reaction product of SK-1, has also been implicated in controlling mycobacterial infection (17). Pathogenic mycobacteria inhibit both SK-1 enzymatic activity as well as its translocation toward phagolysosomal membrane, which prevents the maturation of phagolysosomal compartment (Figure 2) and secure survival of mycobacteria in inflammatory macrophages. Reduced serum titer of S1P in patients with pulmonary tuberculosis provides clinical evidence of such inhibition of SK-1 enzymes by mycobacteria (18). It could be due to the metabolism of S1P into ceramide in macrophages; however, it needs further investigation. S1P triggers multiple signaling pathways, including Ca++ mobilization from ER and the activation of phospholipases, such as phospholipase D, whose antimicrobial activity has been reported (19–21). Since sphingolipids and their derivatives have recently emerged as next generation drug targets for controlling infectious and inflammatory disease; therefore, this review is focused on their dual fate in controlling respiratory infections especially mycobacterial infection.

## Sphingolipids in Respiratory Tract Infections

An increased expression of sialylated glycosphingolipids on lung epithelial cells in cystic fibrosis suggest that sphingolipids can integrate into membrane lipids rafts organelle and serve as receptors for bacterial invasion and inflammatory response. *Mycoplasma pneumonia* infection of lungs results in the induction of autoantibodies against glycosphingolipids, suggesting the involvement of sphingolipid in promoting inflammation in lung (22). Adherence of *Pseudomonas aeruginosa* to asialylated gangliosides, which is a type of sphingolipid, is increased on the surface of cystic fibrosis cells and has been associated with the severity of the disease (23). It has also been shown that interaction of *P. aeruginosa* with host epithelial cells activates host acid sphingomyelinase, leading to the generation of membrane bound ceramide, which triggers apoptosis (24, 25) of the host cell. Intriguingly, *P. aeruginosa* also produces and secretes sphingolipid-metabolizing enzymes, phospholipase-C (26), which can synthesize sphingomyelin from ceramide by enhancing the activity of alkaline ceramidase that break down ceramide. It seems that enzyme production by the bacteria and the type of reaction it catalyzes depend on substrate availability or reaction conditions like free sphingosine or ceramide. Compelling evidences demonstrate that certain pulmonary pathogen like *Chlamydia* cause the trafficking of sphingomyelin and S1PR from trans-Golgi apparatus (27, 28) toward their inclusion membrane (29) for securing their intracellular survival, which contribute to immune evade mechanisms of these bacteria. Thus, these models, supporting our hypothesis, demonstrate dual fate of sphingolipids on both host as well as pathogen during infections.

## Role of Sphingolipids in Fungal Pathogenesis

With the exception of *Sphingobacterium, Sphingomonas*, and *Bacteroides*, prokaryotic cells do not contain their own sphingolipids. However, protozoan and fungal pathogens contain sphingolipids and are found to be involved in their pathogenicity (30). Fungal sphingolipids are important for engulfment and subsequent phagocytosis by the macrophages (31). The pathogen-derived sphingolipids either compete or modulate the sphingolipid signaling in host and interfere with immune response (32). Since last decade, fungal sphingolipids have emerged as a potential target for new antifungal drugs. The first ever evidence that fungi contain sphingolipids came from *Candida albicans*, which was first shown to produce glucosylceramides (32, 33). Recently, it has been shown that *Cryptococcus neoformans*, an opportunistic pathogen, produces inositol-phosphoryl ceramide synthase that plays a key role in virulence of *the pathogen* (34). Downregulation of the enzyme in *C. neoformans* strains has growth deficits when inside alveolar macrophages (31). Other than this, selective stimulation of S1PR2 in host has also shown to confer protection against pathogenesis of this fungus indicated therapeutic potential of S1PR (35) in controlling pathogenesis of this fungus. Other fungal species like *Aspergillus niger* is responsible for invasive pulmonary aspergillosis and also produce specific sphingolipids that play role in pathogenicity (36, 37), raising further scope of targeting these sphingolipids in controlling the pathogenesis of this fungus, which is one of the best known opportunistic fungal pathogen in immunocompromised patients.

## Sphingolipids in Mycobacterial Disease

Mycobacterial infections represent the third major cause of worldwide annual mortalities and have raised serious concerns about the development of effective therapies for controlling infection. Sphingosine kinase-1 is a critical enzyme of sphingolipid metabolism and mediates mycobacteria-induced inflammatory responses in macrophages (38). In line with this, our novel and pioneer study (16) has demonstrated that SK-1 not only orchestrates mycobacterial infection-induced innate immune response but also affords optimum defense against mycobacterial infection in macrophages. Our unpublished data suggest that boosting S1P level pharmacologically in host may offer therapeutic benefit in managing mycobacterial disease. However, among various species of mycobacteria, pathogenic species of mycobacteria particularly MDR/MTR strains contain their own type seventh secretion system, which they use to exploit macrophage defense mechanism for their replication (39–41). Kusner and Russell’s groups have already indicated the significance of ceramide/sphingolipid in establishing mycobacterial persistency (42, 43), which may contribute to the drug resistance. Among those, mycobacteria-induced host sphingolipids (mainly ceramide) are anticipated to confer drug resistance in rifampin-resistant clinical isolate of TB. This could be due to S1P-pAKT-mediated upregulation of mTOR signaling in macrophages (44, 45) by various mycobacterial secretory components.

## Clinical Perspectives

We and other have demonstrated the potential of SK-1/S1P in reducing mycobacterial growth in preclinical models (17), which raised the hope of exploring sphingolipid mimetic as a potential approach for controlling infection. However, it still remains a challenge to predict the outcome of sphingolipid-directed therapeutics in clinical cases like non-reactive and/or extrapulmonary TB cases, which are manifested with aberrant pathology and are usually refractory for immune stimulation. First-generation TB drugs are used routinely for the management of acute TB cases where bacteria display normal pathophysiology, and it is expected that enhancing host sphingolipid signaling or metabolism may afford defense in host against acute infection. However, during chronic or persistent or non-reactive TB, enhanced pulmonary titer of ceramide/sphingolipids in hitratho would oppose immune attack mechanisms and would promote bacterial persistency by their virtue of promote hypoxia. Other mechanisms, which could favor mycobacterial survival either separately or in concert, could include sphingolipid-driven polarization of effector T cells and M1 macrophages toward Treg (46–48) and M2 macrophages (49, 50), which are refractory in nature and promote anti/sterile inflammatory response around granuloma. Under such cases, employing sphingolipids inhibitors in combination of TB drugs is believed to help in breaking mycobacterial persistency and ameliorating rifamycin resistance (51–53), which is still a major challenge for preventing relapse. Exploiting such dual specificity of both sphingolipids mimetics/inhibitors certainly represents novel and future approaches for managing respiratory infection like TB, and this is anticipated that such pharmacological interventions may also reduce the risk of developing lung cancer in TB patients.

## Conclusion and Future Prospective

Among all sphingolipids, ceramide has been the major cause for inflammation and cell death in lungs. However, other sphingolipid metabolites especially S1P and C1P have effects opposite to the ceramide, thus reducing inflammation and apoptosis of cells. It is intriguing to note that the above-reported data suggest that pathologies associated with Cystic fibrosis (CF), Chronic obstructive pulmonary discease (COPD), Respiratory dystress syndrome (RDS), and respiratory tract infections, where inflammation contributes the most, all depend on a balance between ceramide and S1P/C1P concentrations in the lung. More specifically, it is the ratio of ceramide versus S1P/C1P, which determines the extent of inflammation in lungs. Sphingolipids and its metabolites represent a promising drug targets, and within the lung, ceramide metabolism represents a key target for controlling inflammation. However, better understanding of sphingolipid-mediated pathologies and how sphingolipid metabolism can be modified to benefit the host may give a new insight into new therapeutic strategies and can provide alternative or adjuvant to existing therapeutic approaches for managing respiratory syndromes.

## Author Contributions

Both authors have contributed equally to this work and approved it for publication.

## Conflict of Interest Statement

The authors declare that the research was conducted in the absence of any commercial or financial relationships that could be construed as a potential conflict of interest.
